# Long-Term Knowledge Retention of Biochemistry Among Medical Students in Riyadh, Saudi Arabia: Cross-Sectional Survey

**DOI:** 10.2196/56132

**Published:** 2024-12-16

**Authors:** Nimer Mehyar, Mohammed Awawdeh, Aamir Omair, Adi Aldawsari, Abdullah Alshudukhi, Ahmed Alzeer, Khaled Almutairi, Sultan Alsultan

**Affiliations:** 1 College of Science and Health Professions, King Saud bin Abdulaziz University for Health Sciences (KSAU-HS), Riyadh, Saudi Arabia; 2 King Abdullah International Medical Research Center (KAIMRC), Riyadh, Saudi Arabia; 3 Ministry of National Guard - Health Affairs (MNGHA), Riyadh, Saudi Arabia; 4 College of Dentistry, King Saud bin Abdulaziz University for Health Sciences (KSAU-HS), Riyadh, Saudi Arabia; 5 College of Medicine, King Saud bin Abdulaziz University for Health Sciences (KSAU-HS), Riyadh, Saudi Arabia

**Keywords:** biochemistry, knowledge, retention, medical students, retention interval, Saudi Arabia

## Abstract

**Background:**

Biochemistry is a cornerstone of medical education. Its knowledge is integral to the understanding of complex biological processes and how they are applied in several areas in health care. Also, its significance is reflected in the way it informs the practice of medicine, which can guide and help in both diagnosis and treatment. However, the retention of biochemistry knowledge over time remains a dilemma. Long-term retention of such crucial information is extremely important, as it forms the foundation upon which clinical skills are developed and refined. The effectiveness of biochemistry education, and consequently its long-term retention, is influenced by several factors. Educational methods play a critical role; interactional and integrative teaching approaches have been suggested to enhance retention compared with traditional didactic methods. The frequency and context in which biochemistry knowledge is applied in clinical settings can significantly impact its retention. Practical application reinforces theoretical understanding, making the knowledge more accessible in the long term. Prior knowledge (familiarity) of information suggests that it is stored in long-term memory, which makes its retention in the long term easier to recall.

**Objectives:**

This investigation was conducted at King Saud bin Abdulaziz University for Health Sciences in Riyadh, Saudi Arabia. The aim of the study is to understand the dynamics of long-term retention of biochemistry among medical students. Specifically, it looks for the association between students’ familiarity with biochemistry content and actual knowledge retention levels.

**Methods:**

A cross-sectional correlational survey involving 240 students from King Saud bin Abdulaziz University for Health Sciences was conducted. Participants were recruited via nonprobability convenience sampling. A validated biochemistry assessment tool with 20 questions was used to gauge students’ retention in biomolecules, catalysis, bioenergetics, and metabolism. To assess students’ familiarity with the knowledge content of test questions, each question is accompanied by options that indicate students’ prior knowledge of the content of the question. Statistical analyses tests such as Mann-Whitney *U* test, Kruskal-Wallis test, and chi-square tests were used.

**Results:**

Our findings revealed a significant correlation between students’ familiarity of the content with their knowledge retention in the biomolecules (*r*=0.491; *P*<.001), catalysis (*r*=0.500; *P*<.001), bioenergetics (*r*=0.528; *P*<.001), and metabolism (*r*=0.564; *P*<.001) biochemistry knowledge domains.

**Conclusions:**

This study highlights the significance of familiarity (prior knowledge) in evaluating the retention of biochemistry knowledge. Although limited in terms of generalizability and inherent biases, the research highlights the crucial significance of student’s familiarity in actual knowledge retention of several biochemistry domains. These results might be used by educators to customize instructional methods in order to improve students’ long-term retention of biochemistry information and boost their clinical performance.

## Introduction

The knowledge presented within the context of basic science establishes a solid base for understanding biological system activity from the biomolecular to the organismal level under various normal and pathological conditions. Many educators believe that during the early stages of medical education, this base of knowledge is necessary to maximize initial learning of clinical medicine [1]. Eventually, basic science knowledge will be conceptually integrated with clinical knowledge, giving students a deeper comprehension of diseases’ mechanisms and better diagnosis skills [2]. Later, during professional practice years, this integrated framework of basic and clinical knowledge continues to help and improve medical doctors’ diagnostic proficiency, particularly when dealing with new or complex medical conditions [3,4].

On the contrary, a considerable number of studies confirm that practicing medical doctors lose a significant portion of the basic science information they learned during early medical education stages [5-10]. One way to categorize the period between a participant’s exposure to information and being tested for its retention is through the retention interval (RI). In a study in 1998, Ellis et al [11] found that students lost about one-third of nonuse information after 1 year and about half after 2 years. Also in 1981, Rico et al [12] reported that two-thirds of knowledge was lost after 8 years of nonuse. Moreover, many studies emphasize that basic sciences do not significantly improve medical students’ clinical proficiency due to the huge amount of theoretical information, lack of connection to the clinical field, and passive ways of information delivery. Many medical students consider basic sciences as a burden rather than a useful subject [6,13-16]. Collectively, the outcomes of these studies support the view of a reduced contribution of basic science to medical curricula [11]. As a result, more educators see that integrated and self-directed educational approaches within a problem-based curriculum are the best methods to introduce basic sciences into medical education [13-15].

Nevertheless, the controversy continues as the same results can be used to prove the opposite point of view. This team of medical educators stresses that the basic science knowledge is not completely forgotten and can be efficiently retrieved even a long time after finishing medical school [13,17,18]. Seemingly, individuals tend to underestimate how much knowledge they retain after long periods of nonuse [19]. However, when correctly measured, the loss of basic science information is not a negatively proportional process, but rather a gradually decelerating nonlinear process [20], which suggests that a portion of information is never completely lost but rather transformed to a permanent storage state (permastore memory) and remains functionally active and influential in medical doctors’ professional practice as they can be easily recalled even after long times of nonuse [21]. For this reason, the same studies used to emphasize loss of unrehearsed knowledge can be equally used to prove the retention of a considerable portion of unrehearsed knowledge. In other words, students were able to retain 40%‐50% of unrehearsed knowledge after 2 years [11]. Similarly, medical doctors retained one-third of their basic science knowledge after 8 years of nonuse [12]. In a third study, 15%‐20% of basic sciences information was successfully retrieved even after 25 years of nonuse [11]. Many educators argue that knowledge cannot be completely forgotten but rather, it becomes temporarily difficult to retrieve. Therefore, changes in medical education should be directed towards adapting teaching methods that are shown to increase knowledge retrievability [11].

Many studies agree that biochemistry, among other basic sciences, has a low long-term retention [21-23]. Moreover, building on the previously mentioned impact of the duration of nonuse on retention [24], many studies indicate a strong association between poor biochemistry retention and the low perception of clinical prevalence of biochemistry [23]. Moreover, this low perception of clinical prevalence of biochemistry is also associated with low clinical performance [25-28]. Despite this apparent characteristic of quick loss of biochemistry information after graduation, a study conducted on medical graduates during their internship training indicated that 64% of medical graduates retained 40%‐60% of biochemistry knowledge [29]. This study suggests that medical graduates remained familiar with different aspects of biochemical information even after they had stopped using this knowledge for a long time. The finding of this study aligns with other research, which indicates that previously known knowledge (familiar) is stored in long-term memory. High-familiarity knowledge makes it easier to recall compared with moderately familiar knowledge [30]. Deeper insights of long-term retention studies can be gained by looking into the impact of familiarity on knowledge retention. For that reason, this study aims to assess the association between the familiarity of biochemistry information and its retention among medical students at King Saud bin Abdulaziz University for Health Sciences (KSAUHS) in Riyadh, Saudi Arabia, following the completion of their biochemistry course.

## Methods

### Study Design and Area and Settings

KSAUHS, Riyadh, Saudi Arabia, has a total of 2000 students from both colleges of medicine and dentistry. Students of the third, fourth, fifth, and sixth academic years were directly approached during their classes on several occasions to participate in the study. A total of 240 students have agreed to participate in this study. Students were recruited from September 1, 2022, until September 15, 2022, by nonprobability convenience sampling and were asked to answer this cross-sectional correlational survey.

### Ethical Considerations

All the procedures of this study have been approved by the King Abdullah International Medical Research Center institutional review board (IRB/1298/22) to ensure its compliance with King Abdullah International Medical Research Center ethical standards of research involving human participants. The participation was entirely voluntary; thus, no formal consent form was required. Students were initially provided with a clear explanation of the study’s purpose, procedures, and the use of the collected data. They were assured that their responses will be anonymous and confidential.

### Identification of Study Participants

The study included years 3 (n=99), 4 (n=42), 5 (n=36), and 6 (n=49) students from both medicine and dentistry colleges. Years 1 and 2 students were excluded due to either not starting or not finishing the course yet as the biochemistry course is taught in the second semester of the second foundation year. It is a 4-hour credited course that is an introductory-level course designed to provide basic foundations in biochemistry. The time span between the participants starting biochemistry and the time they took the test ranged from 1 year for third-year students (2022), 2 years for fourth-year students (2021), 3 years for fifth-year students (2020), and 4 years for sixth-year students (2019). These different RIs from these 4 groups were then used to compare the retention of knowledge in those participants. It is worth noting that in 2019 delivery of the biochemistry course was changed to online instead of on campus due to the COVID-19 pandemic. Identification parameters that were used included age, gender, major, class, teaching role, grade point average (GPA), and grade. A teaching role means that the participant had any chance of giving students from earlier years biochemistry classes. This is important because this can have an impact on the retention of the biochemistry knowledge.

### Sample Size Calculation

Assuming a correlation coefficient of 0.20 between knowledge retention and RI, a power of 0.80, and a significance level (α) of .05, the sample size will be at least 194 participants.

### Tool Description

In this study, we used a biochemistry knowledge retention assessment tool that was developed by Dr Eugène J F M Custers in 2010 with some modifications (Table 1). Composed of 20 short answer questions, this tool was designed to effectively measure the understanding of biochemistry among medical students. The tool consists of questions that cover the following 4 key domains: biomolecules (n=8), catalysis (n=5), bioenergetics (n=2), and metabolism (n=5). This tool was specifically developed to capture a comprehensive understanding of these crucial areas in biochemistry. The development process involved a thorough selection of items that are representative of the essential knowledge in each domain. To assess participants’ familiarity with the content of each question, at the end of each question, participants were asked to select 1 of 4 options: “Unknown,” “Known but forgotten,” “Known but unseen since completing the biochemistry course,” and “Known and seen after completing the biochemistry course.” The original tool was published and validated. Before taking the test, students were asked to fill in information about their gender, major, class, teaching experience, GPA, and their grade in the introductory biochemistry course offered on second year.

**Table 1.** Biochemistry knowledge retention assessment test.

Domain and question		Answer
**Biomolecules**		
	Q 1: What is the name of a polymer chain of amino acids and what is the name of the covalent bond that connects them?	(a) Polypeptide, (b) peptide bond
	Q 2: Name 4 amino acids.	Any 4 of the 20 amino acids (full name, 3-letter, and 1-letter abbreviations are all accepted)
	Q 3: Name 2 disaccharides.	Any 2 of the most popular disaccharides (eg, sucrose, lactose, maltose, trehalose, and cellobiose)
	Q 4: Name a polysaccharide found in plant cell wall and humans cannot digest.	Cellulose (fiber)
	Q 5: Name the 4 nitrogen bases found in DNA. In double-strand DNA.	Adenine (A), guanine (G), thymine (T), and cytosine (C)
	Q 6: Name 3 types of RNA.	mRNA, tRNA, and rRNA (other less-known types are also acceptable)
	Q 7: Which cellular organelle (component) is mainly made of phospholipids?	Membranes
	Q 8: Storage lipids such as fats and oils are chemically made of 3 fatty acids connected to 1 molecule by a glycosidic bond (linkage). What is this molecule?	Glycerol
**Catalysis**		
	Q 9: Enzymes specificity can be explained by 2 models. First: lock and key model. What is the name of the second model?	Induced fit
	Q 10: An equation relates the change of reaction rate (v0) in response to the change in the substrate concentration [S]. It is used to estimate the maximum reaction rate (V_max_) and substrate binding affinity (KM). These 2 parameters are used to describe the enzymatic reactions (ie, each enzyme has a unique V_max_ and KM). What is the name of this equation?	Michaelis-Menten equation
	Q 11: What is the name of the molecule that resembles the original substrates and binds to the catalytic site of the enzyme and thus preventing the original substrate binding?	Competitive inhibitor
	Q 12: What is the name of the process that uses energy and specialized transport proteins to move ions or molecules across a cellular membrane from a region of low concentrations to another region of higher concentrations (against the gradient)?	Active transport
	Q 13: What do you call a protein located on the surface of a cell that binds to a specific substance and causes a specific response in the cell?	Receptor
**Bioenergetics**		
	Q 14: What is the most abundant energy carrier molecule in cells?	Adenosine triphosphate
	Q 15: When the DG of chemical reaction is negative, then this reaction happens by itself (spontaneously), What is DG?	Free energy
**Metabolism**		
	Q 16: Name 2 hormones released from the pancreas and maintain glucose levels in human blood.	Insulin and glucagon
	Q 17: In normal conditions, name one human organ that is mainly or entirely dependent on glucose as a source of energy?	Red blood cells or brain
	Q 18: In normal conditions, name 1 human tissue that is responsible for fat synthesis (lipogenesis).	Liver or adipose tissues
	Q 19: What is the name of the glucose breakdown pathway to pyruvate?	Glycolysis
	Q 20: When the first reaction of a multistep metabolic pathway (pathways of several reactions) is inhibited by the product of the last reaction, what this kind of inhibition is called?	Feedback inhibition

### Data Collection Process

The test forms were handed in-person to the participants and conducted in auditoriums after they have finished their sessions (lectures, problem-based learning sessions, etc) and they were asked to complete the form without using any books, phones, or any consultations. For every group, there was an examination supervisor to make sure no cheating is taking place. After checking their consent to take the survey, every group was given 30 minutes to finish the questionnaire and then hand the form back to the supervisor.

### Statistical Analysis Plan

The research aims to assess the enduring grasp of biochemistry among medical students at KSAUHS in Riyadh, Saudi Arabia. The study involved a comprehensive statistical analysis of the collected data, using both descriptive and inferential statistics. Analyzing data involved numerical and graphical presentations. A set of 20 biochemistry questions was used, each requiring both a factual answer and a descriptive statement of whether the students were already familiar with the question beforehand. Due to variables not meeting parametric conditions, nonparametric tests were used for comparisons. To explore variations in total answer scores based on sociodemographic factors, statistical tests such as the Mann-Whitney *U* test, Kruskal-Wallis test, and Spearman rank test were applied. Statistical significance was established at a *P* value of .05 or lower with a 95% CI. SPSS software (version 27.0.0; IBM Corp) was used for all statistical analyses. The questionnaire in the study comprised 20 biochemistry questions, distributed across biomolecules (n=8), catalysis (n=5), bioenergetics (n=2), and metabolism (n=5) topics. During analysis, proportions of correct and partially correct answers were combined in 1 group, “Correct” and coded with 1. Incorrect answer proportions were considered as 1 group “Incorrect” and coded with 0. Means of correct and incorrect proportions were calculated. Means of correct answers proportions were used to indicate students’ retention levels for each topic. On the other hand, the proportions of students who described their prior knowledge of the content of a given question as “Uknown” and those who described their prior knowledge as “Known but forgotten” were combined in 1 group, “Unfamiliar.” Proportions of students’ prior knowledge descriptions as “Known but unseen since completing the biochemistry course” and “Known and seen after completing the biochemistry course” were combined in 1 group, “Familiar.” During the analysis, the unfamiliar group was given a 0 code, while the familiar group was given a 2 code. Mean scores of familiar and unfamiliar groups were calculated. Means of familiar answer proportions were used to indicate students’ level of familiarity for each topic.

## Results

As shown in Table 2, a total number of 240 students from KSAUHS in Riyadh participated, with an average age of 21.29 years. The majority were male studying medicine and dentistry across various levels and achieving a GPA above 4.5. Concerning biochemistry grades, a notable number received A+, A, and B+ grades. In addition, the majority did not have any teaching roles. All the participants are students (n=240, 100%) and from KSAUHS, Riyadh, Saudi Arabia (n=240, 100%).

**Figure 1.**
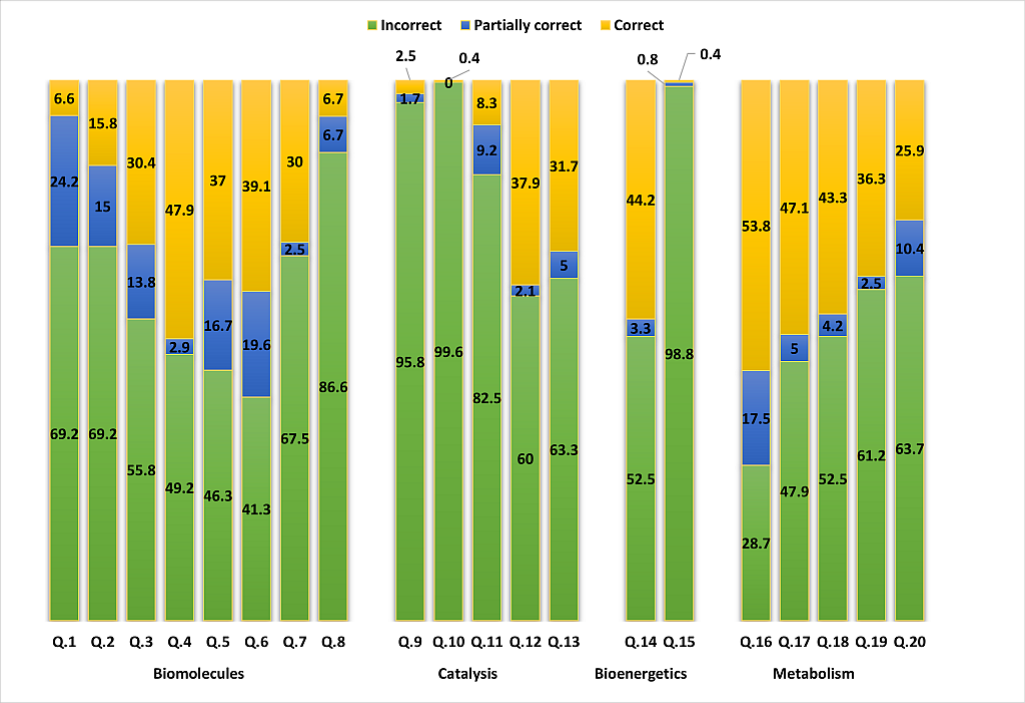
Actual students’ answers (correct, partially correct, or incorrect) to different biochemistry knowledge domains: biomolecules (questions 1‐8), catalysis (questions 9‐13), bioenergetics (questions 14 and 15), and metabolism (questions 16‐20).

Figure 1 provides a detailed explanation of the distribution of students correct, partially correct and incorrect answers for each biochemistry question. It is evident that certain questions were largely unknown to most students, resulting in predominantly incorrect answers. For example, questions 10 (99.6%), 15 (98.8%), 9 (95.8%), 8 (86.7%), 11 (82.5%), and questions 1 and 2 (69.2%) received predominantly incorrect responses. Conversely, only few questions were relatively familiar to students, leading to higher rates of correct answers. For instance, question 16 (53.8%), question 4 (47.9%), question 17 (47.1%), question 14 (44.2%), and question 18 (43.3%) received relatively higher rates of correct responses.

**Table 2.** Sociodemographic characteristics of participants.

Characteristic		Value
Age in years, mean (SD)		21.29 (1.73)
**Gender, n (%)**		
	Male	206 (85.8)
	Female	33 (13.8)
**Major, n (%)**		
	Medicine	201 (83.8)
	Dentistry	32 (13.3)
**Class, n (%)**		
	3	99 (41.3)
	4	42 (17.5)
	5	36 (15.0)
	6	49 (20.4)
**Teaching role, n (%)**		
	Yes	3 (1.3)
	No	219 (91.3)
**GPA** ^a^ **, n (%)**		
	3‐3.99	17 (7.1)
	4‐4.49	63 (26.3)
	≥4.5	142 (59.2)
**Biochemistry grade, n (%)**		
	A+	64 (26.7)
	A	47 (19.6)
	B+	44 (18.3)
	B	20 (8.3)
	C+	23 (9.6)
	C	5 (2.1)
	D+	4 (1.7)
	D	3 (1.3)

a GPA: grade point average.

**Figure 2.**
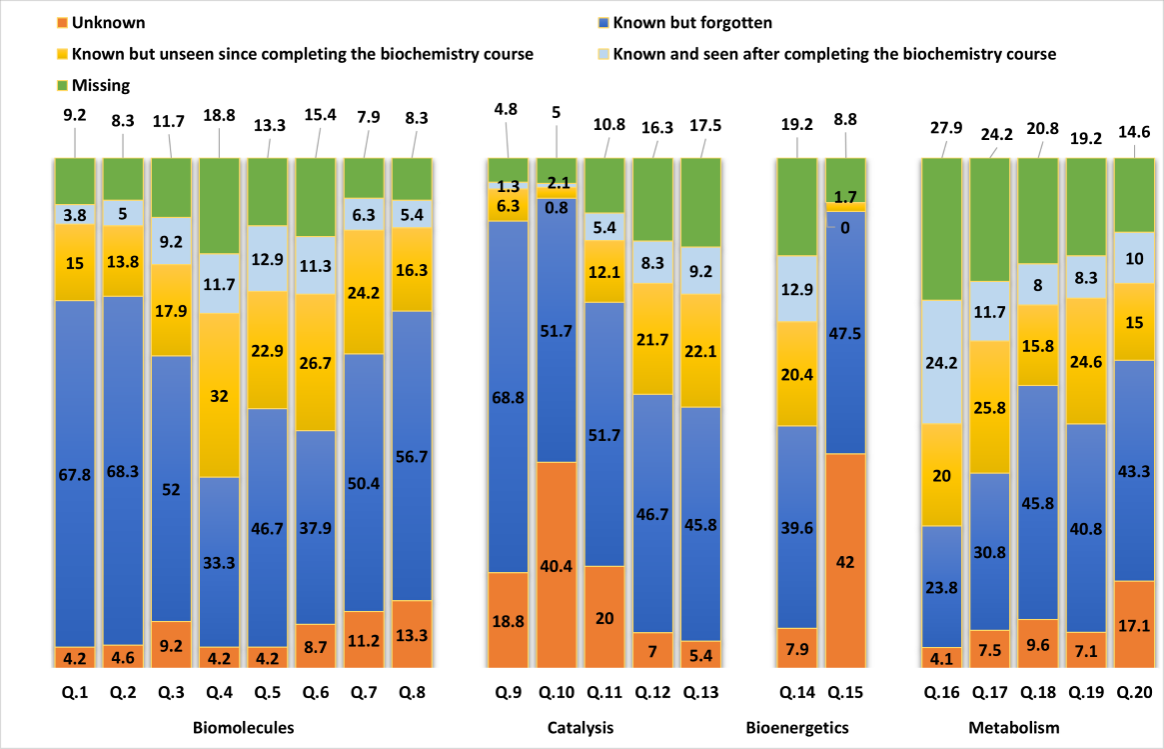
Students’ familiarity with question content (unknown, known but forgotten, known but unseen since completing the biochemistry course, known and seen after completing the course, and missing) of different biochemistry knowledge domains: biomolecules (questions 1‐8), catalysis (questions 9‐13), bioenergetics (questions 14 and 15), and metabolism (questions 16‐20).

The findings highlighted in Figure 2 align consistently with those in Figure 1. Questions that attracted more incorrect responses in Figure 1 also collected a higher frequency of students stating, “I never knew this” or “I knew this but forgot it” in response to those questions. When organized in ascending order, the combined proportions of these 2 descriptive responses were 70% for question 8, 71.7% for question 11, 72.1% for question 1, 72.9% for question 2, 87.6% for question 9, and 92.1% for question 10. This correspondence between Figures1  2 confirms the strong correlation between factual students’ answers and subjective descriptive responses about their familiarity of the content shown in Table 3.

**Table 3.** Correlation analysis (Spearman rank test, *P* value) between correct answer proportion and familiar response proportion of each biochemistry knowledge domain.

Variable	Correct	Familiar	*r*	*P* value^a^
Biomolecules (Q1-Q8), mean^b^	39.5	29.3	0.491	.001
Catalysis (Q9-Q13), mean^b^	19.9	17.8	0.500	.001
Bioenergetics (Q14-Q15), mean^b^	24.4	17.5	0.528	.001
Metabolism (Q16-Q20), mean^b^	49.2	32.7	0.564	.001

a Spearman rank test.

b Adjusted mean.

As previously mentioned in the Methods section, the proportions of “correct” and “partially correct” answers of each domain were combined in 1 group, “Correct.” Adjusted means of combined proportions were calculated. On the other hand, the proportions of “known but unseen since finishing the course” and “known and seen after finishing the course” were combined in 1 group, “Familiar.” Adjusted means of combined groups were calculated. The correlation between adjusted means of correctly answered questions identified as “Correct” and question content familiarity identified as “Familiar” was computed for each domain using Spearman rank test (Table 3). The table illustrates notably significant strong correlations between the adjusted means of correct students’ answers and students’ familiarity. Specifically, correlation coefficients of 0.49 were observed for the biomolecules, 0.50 for catalysis, 0.52 for bioenergetics, and 0.564 for metabolism. This reveals that students who selected accurate answers also demonstrated familiarity with the related information, and, conversely, those who were not familiar with the content tended to provide wrong responses.

Table 4 elaborates on the outcomes shown in Table 2. Through chi-square tests or Fisher exact tests, significant associations have been established between the actual and descriptive answers for each question. As previously mentioned in Methods section, the numbers and frequencies of students’ answers were grouped into “Correct” and “Incorrect” groups. Also, the numbers and frequencies of students’ prior knowledge responses were grouped into “Familiar” and “Unfamiliar.” Questions recognized as familiar by students were correlated with higher rates of correct responses, while those recognized as unfamiliar were linked with a higher number of incorrect answers.

The potential associations between students’ correct answers, familiarity responses, and various sociodemographic factors were explored (Table 5). Students in the third level scored notably higher for correct answers than those in other levels. This could be attributed to their recent exposure to biochemistry in the second medical grade curriculum, leading to better retention of information. Similarly, students with a GPA exceeding 4.5 also displayed a higher rate of correct answers. Furthermore, students attaining A and A+ grades in biochemistry achieved the highest mean scores for correct answers. However, variables such as age, gender, field of study, and having a teaching role did not significantly impact students’ actual knowledge of biochemistry. The potential associations between students’ familiarity with the questions and various sociodemographic factors were examined. Notably, GPA emerged as the sole factor influencing their familiarity score. Students with a GPA more than 4.5 demonstrated the highest mean score of familiarity. Conversely, variables such as age, gender, field of study, level of study, biochemistry grade, and having a teaching role did not notably impact students’ familiarity with the biochemistry question content.

**Table 4.** Associations (chi-square and Fisher exact tests *P* values) between students’ answer proportions and familiarity response proportions of biochemistry knowledge test questions.

Domain and question		Familiar		Unfamiliar		*P* value
		Incorrect, n (%)	Correct, n (%)	Incorrect, n (%)	Correct, n (%)	
**Biomolecules**						
	Q1	11 (24.4)	34 (75.6)	145 (83.8)	28 (16.2)	.001
	Q2	11 (24.4)	34 (75.6)	145 (82.9)	30 (17.1)	.001
	Q3	9 (13.8)	56 (86.2)	117 (79.6)	30 (20.4)	.001
	Q4	27 (25.7)	78 (74.3)	75 (83.3)	15 (16.7)	.001
	Q5	10 (11.6)	76 (88.4)	92 (75.4)	30 (24.6)	.001^a^
	Q6	10 (11.0)	81 (89.0)	79 (70.5)	33 (29.5)	.001
	Q7	18 (24.7)	55 (75.3)	135 (91.2)	13 (8.8)	.001
	Q8	33 (63.5)	19 (36.5)	159 (94.6)	9 (5.4)	.001
**Caralysis**						
	Q9	11 (61.1)	7 (38.9)	208 (99.0)	2 (1.0)	.001
	Q10	6 (85.7)	1 (14.3)	221 (100.0)	0 (0.0)	.03^a^
	Q11	16 (38.1)	26 (51.9)	163 (94.8)	9 (5.2)	.001^a^
	Q12	16 (22.2)	56 (77.8)	115 (89.1)	14 (10.9)	.001
	Q13	26 (34.7)	49 (65.3)	107 (87.0)	16 (13.0)	.001
**Bioenergetics**						
	Q14	13 (16.3)	67 (83.7)	96 (84.2)	18 (15.8)	.001
	Q15	2 (50.0)	2 (50.0)	214 (99.5)	1 (0.5)	.001^a^
**Metabolism**						
	Q16	4 (3.8)	102 (96.2)	53 (79.1)	14 (20.9)	.001
	Q17	16 (17.8)	74 (82.2)	80 (87.0)	12 (13.0)	.001
	Q18	5 (8.8)	52 (91.2)	107 (80.5)	26 (19.5)	.001
	Q19	17 (21.5)	62 (78.5)	110 (95.7)	5 (4.3)	.001
	Q20	8 (13.3)	52 (86.7)	133 (91.7)	12 (8.3)	.001

a Fisher exact test.

**Table 5.** Associations (Spearman rank test, Mann-Whitney *U* test, and Kruskal-Wallis test *P* values) between correct answer proportions, familiarity response proportions, and sociodemographic characteristics.

Characteristic		Correct		Familiar	
		Value	*P* value	Value	*P* value
Age (years), *r*		−0.09	.17^a^	−0.05	.45^a^
**Gender, mean (SD)**		—^b^	28^c^	—	.46^c^
	Male	6.99 (4.71)		22.62 (7.99)	
	Female	7.91 (4.82)		21.09 (9.34)	
**Major, mean (SD)**		—	.37^c^	—	.48^c^
	Medicine	6.95 (4.69)		22.64 (7.97)	
	Dentistry	7.69 (4.72)		21.09 (9.49)	
**Class, mean (SD)**		—	.001^d^	—	.10^d^
	3	8.58 (4.86)		23.80 (8.64)	
	4	3.98 (3.89)		20.79 (6.94)	
	5	7.03 (5.05)		21.67 (8.83)	
	6	7.18 (3.61)		22.37 (7.58)	
**Teaching role, mean (SD)**		—	.21^c^	—	.60^c^
	Yes	10.33 (4.16)		24.33 (13.86)	
	No	6.95 (4.71)		22.24 (8.11)	
**GPA^e^, mean (SD)**		—	.001^d^	—	.01^d^
	3‐3.99	3.76 (2.25)		18.47 (4.25)	
	4‐4.49	5.78 (4.09)		21.95 (6.73)	
	≥4.5	8.17 (4.84)		23.22 (8.83)	
**Biochemistry grade, mean (SD)**		—	.001^d^	—	.14^d^
	A+	9.92 (4.79)		24.53 (9.17)	
	A	7.02 (4.25)		22.02 (7.87)	
	B+	6.64 (4.34)		23.27 (7.97)	
	B	5.00 (3.58)		22.00 (5.75)	
	C+	4.78 (4.23)		21.39 (7.07)	
	C	3.80 (2.58)		22.00 (4.84)	
	D+	3.00 (2.16)		17.25 (2.63)	
	D	4.33 (3.05)		22.00 (7.81)	

a Spearman rank test.

b Not applicable.

c Mann-Whitney *U* test.

d Kruskal-Wallis test.

e GPA: grade point average.

## Discussion

### Principal Findings

Recently, subjects such as biochemistry and molecular biology have achieved significant success. This is because biochemical concepts and techniques have become essential components of research in various fields, such as genetics, pharmacology, microbiology, endocrinology, immunology, nutrition, pathology, and other clinical disciplines. During the preclinical period of the medical course, biochemistry plays a crucial role in comprehending the structure and function of many biomolecules in the human body, both in terms of disease and health. This study used various questions to evaluate the long-term retention of biochemistry knowledge skills among medical students in KSAUHS, Riyadh, Saudi Arabia. The findings of this indicate that medical students showed different levels of retention observed across various biochemistry domains and specific questions. Some topics showed higher retention than others. Students displayed varying levels of familiarity with different biochemistry concepts, leading to different correctness rates in their answers. This suggests that some of the participants see biochemistry as a challenging topic or as having little relevance to medical education rather than considering it a crucial component of their medical training. The study highlights the idea that while there is a decrease in retention, a significant amount of the taught information remains stored and can be retrieved. This emphasizes the need to use measures to improve the accessibility of stored knowledge [31]. The reason behind year 4 student having statistically significant lower grade compared with the other years could be attributed to how the delivery of biochemistry knowledge was switched from in-campus to web-based sessions due to COVID-19 pandemic.

The research conducted by Sé et al [18] examined the perceptions of King’s College medical graduates on how effectively their undergraduate degree prepared them for medical practice. The study analyzed 5 cohorts of graduates. Specifically, they were requested to assess the degree of accuracy of the information presented in various preclinical courses. The study’s most notable finding is the high percentage of physicians (79.5%) who thought their “traditional” basic science course included “too much” biochemistry [18]. While these kinds of investigations do reveal actual problems, they also seem to bring up many more questions than they resolve. What about physicians who learn biochemistry in an “integrated” class, a “Problem-based Learning” program, or a hybrid system? Doctors who have received training in the integrated system have not been the subjects of any research. Linear data, however, suggest that students learn much less biochemistry in problem-based learning courses than in traditional ones. Many people feel that traditional medical education should focus more on biochemistry. The doctors who participated in the King’s research went on to say that they were taught an overwhelming amount of biochemistry that did not have any effect on real-world practice, and that this made them study too much for tests [18,29].

Statistical associations highlight the influence of various sociodemographic factors on biochemistry retention. Consistent with prior research conducted by Ling et al [22], statistical significance emerges between higher GPAs and improved retention, indicating academic performance as a robust predictor of long-term knowledge recall. Similarly, statistically significant associations between superior biochemistry grades and better retention reinforce the established link between course performance and sustained knowledge retention. However, variables such as age, gender, and field of study did not exhibit statistically significant impacts on students’ actual biochemistry knowledge, contrasting earlier research attributing more influence to these factors [22,32,33].

Statistics reveal a striking correlation between students’ familiarity with biochemistry content and their ability to provide correct answers, highlighting the role of prior knowledge in evaluating knowledge retention. The strong correspondence emphasizes the significance of long-term memory in gauging knowledge retention levels [30]. The observed pattern, where students unfamiliar with the content demonstrated lower rates of correct answers, resonates with previous studies. It substantiates the argument put forth by El-Bab et al [25] indicating that the retention and application of knowledge are contingent upon prior exposure and familiarity. The statistically significant associations, indicated by *P* values across most questions, underline the pivotal link between recognition of biochemistry information and accurate responses. This finding concurs with a study conducted in 2013 supporting the notion that familiarity with subject matter significantly influences students’ performance in related assessments [27]. Notably, questions where students reported higher familiarity with the content (“Familiar”) exhibited substantially better rates of correct answers than questions where students indicated unfamiliarity (“Unfamiliar”). This correlation between recognition and accuracy reinforces the conclusions drawn by Hamza et al [29] emphasizing that exposure and awareness strongly impact students’ ability to recall and correctly apply knowledge.

The study’s statistical findings have substantial ramifications for medical education. The statistical data highlight the ongoing difficulty of maintaining long-term retention and calls for a reassessment of instructional approaches. Methods such as spaced repetition, active learning approaches, and incorporating fundamental sciences into clinical settings, backed by statistical significance, show the potential to strengthen the long-term retention of medical students [34].

### Strengths and Limitations

This study has various strengths and limitations that should be considered. First, it thoroughly explores various facets, including long-term retention, demographic influences, and the correlation between students’ familiarity with the content and actual retention, offering a holistic view of knowledge retention dynamics. Second, it uses a cross-sectional correlational survey, ensuring a robust approach to data collection. The utilization of a validated biochemistry assessment tool enhances the study’s credibility. Third, our study uses suitable statistical analyses such as Mann-Whitney *U* test, Kruskal-Wallis test, Spearman rank test, and chi-square tests, boosting the validity of the conclusions drawn. Fourth, the study’s focus on medical education and its implications for professional practice renders the findings pertinent and potentially impactful in real-world settings.

The limitations of the study included limited generalizability. Findings might be specific to KSAUHS, Riyadh, restricting their applicability to other contexts or regions. Moreover, the use of convenience sampling might introduce biases, affecting the representation of the entire student population. The cross-sectional design provides a single-time assessment, potentially overlooking fluctuations or changes in retention over a longer period. Also, relying on self-reported familiarity with biochemistry content may introduce subjectivity or inaccuracies in evaluating actual retention levels. Future research, supported by robust statistical methodologies, could use diverse assessments and delve deeper into contextual and curriculum-related aspects impacting knowledge retention in biochemistry among medical students.

### Conclusions

This study examines the complex dynamics of how medical students at KSAUHS in Riyadh, Saudi Arabia, retain information in the field of biochemistry. The study emphasizes the important significance of content prior knowledge (familiarity) in measuring information retention. Although there are limits in how widely these results may be applied and various biases that may affect them, they emphasize the need for using customized instructional methods that promote students’ familiarity with the presented knowledge. By addressing these subtle distinctions, there is potential to greatly increase the long-term retention of information. This would provide vital insights to educators who are seeking to optimize medical curricula and boost students’ understanding and practical application of biochemistry in clinical settings.

## Abbreviation

GPA: grade point average
KSAUHS: King Saud bin Abdulaziz University for Health Sciences
RI: retention interval
